# What facilitates a sense of belonging amongst Australian teachers?

**DOI:** 10.1080/00049530.2025.2459190

**Published:** 2025-02-05

**Authors:** Kelly-Ann Allen, Fiona Longmuir, Megan G. Thorn, Ebony Melzak, Emily Berger, Beatriz Gallo Cordoba, Michael Phillips, Andrea Reupert

**Affiliations:** a School of Educational Psychology and Counselling, Faculty of Education, Monash University, Clayton, Australia; b Wellbeing Science Centre, Faculty of Education, University of Melbourne, Parkville, Australia; c School of Education, Culture and Society, Faculty of Education, Monash University, Clayton, Australia; d School of Rural Health, Faculty of Medicine, Nursing and Health Sciences, Monash University, Churchill, Victoria; e Centre for International Research on Education Systems, Mitchell Institute, Victoria University, Melbourne, Australia; f School of Curriculum, Teaching and Inclusive Education, Faculty of Education, Monash University

**Keywords:** Sense of belonging, Australian teachers, teacher shortage, thematic analysis

## Abstract

**Objective:**

Enhancing a sense of belonging among teachers could be key to mitigating global teacher shortages. This study aimed to investigate teachers' sense of belonging in the Australian educational context.

**Method:**

A sample of 3,206 teachers answered a survey with open-ended questions that were analysed using thematic analysis.

**Results:**

The analysis identified six key themes influencing teachers' sense of belonging: Interpersonal relationships (peer relationships, student and parent relationships); Support and collaboration (providing and receiving support; team dynamics and collaborative efforts); Professional and personal growth (influence and contribution to society, professional learning opportunities, individual professional identity and characteristics); Institutional factors (leadership support, positive school environment, employment stability), Motivators (teaching passion, acknowledgment and appreciation); and External networks (engagement in professional networks).

**Conclusions:**

The findings provide insights into strategies for enhancing teacher belonging, with potential implications for improving retention and addressing teacher shortages.

## Key Points

**What is already known about this topic:**

1. The increasing rates of teacher attrition have been identified as a significant issue worldwide, with negative impacts on student outcomes, organisational stability, and the overall educational system. Factors contributing to attrition include high workloads, lack of support, and misalignment of values between teachers and educational institutions.
2. Research has shown that a strong sense of belonging in the workplace is associated with various positive outcomes, including higher productivity, reduced distress, enhanced resilience, and increased intent to stay in the profession.
3. Previous studies have highlighted the importance of interpersonal relationships, particularly with colleagues, in fostering a sense of belonging in the workplace. In educational settings, strong collegial relationships are critical for teacher satisfaction and retention.

**What this topic adds:**

1. This study identifies six key themes that contribute to Australian teachers’ sense of belonging: Interpersonal Relationships, Support and Collaboration, Professional and Personal Growth, Institutional Factors, Motivators, and External Networks.
2. By highlighting the specific factors that enhance teachers’ sense of belonging, this research offers actionable insights for policymakers and educational leaders to develop targeted strategies aimed at improving teacher retention, thereby addressing the teacher shortage crisis.
3. The study reveals the significant role of external professional networks, including unions and online communities, in fostering a sense of belonging among teachers. This expands the understanding of belonging beyond the immediate school environment, emphasising the importance of broader professional engagement.

## Introduction

Belonging is a complex and multifaceted construct that can be conceptualised as the subjective feeling of deep connection with social groups, physical places, and individual and collective experiences that emerges from the interplay of one’s competencies, opportunities, motivations, and perceptions within social and environmental contexts (Allen et al., 2021). Whilst Leary and Baumeister suggest belonging relates closely to social acceptance and interpersonal relationships (Baumeister & Leary, 1995; also see Allen, Berger, et al., 2023), conceptualisations of belonging drawn from Indigenous knowledge systems consider belonging as ontological, extending beyond social situations to encompass connections with Country, place, and land. This Indigenous conceptualisation of belonging is therefore inextricably linked to sovereignty and inseparable from the ongoing impacts of colonisation and dispossession (Moreton-Robinson, 2015; Rey, 2021).

The need to belong is a foundational experience of human existence and in the longer term, considered to be as important to human survival as food, shelter, and water (Baumeister & Leary, 1995; Glasser, 1986) and has been in examined in various contexts including workplaces and schools (Allen et al., 2018; Hagerty et al., 1992). In the realm of teachers’ work, understanding belonging is essential. Rising rates of teacher attrition from the profession in Australia poses a significant challenge to educational quality and organisational stability (Department of Education, 2022; Heffernan et al., 2022). Understanding what fosters teachers’ sense of belonging may be a critical way to address retention. This study investigates the key factors that contribute to Australian teachers’ sense of belonging in their school communities.

### Workplace belonging

The workplace is an important context for belonging research, with many people spending a substantial amount of their lives at work (Barton, 2023; Thissen et al., 2023). Research shows several benefits associated with employees feeling a strong sense of belonging to their workplace, including greater productivity, reduced distress, enhanced resilience levels and increased intent to stay in the role (Blau et al., 2023; Shakespeare-Finch & Daley, 2017). Conversely, research that examined constructs often associated with a lack of belonging within workplaces, such as social exclusion or (un)belonging have found a negative impact on the efficiency and culture of the workplace through its association with poorer quality teamwork, poorer employee physical wellbeing (e.g., poor sleep), and intent to remain in the role (e.g., Barton, 2023; Horak & Suseno, 2022; Pereira et al., 2012; Scott et al., 2014; Thissen et al., 2023). Yet, many of these outcomes could be mediated by actively promoting belonging. Thissen et al. (2023) found that facilitating belonging within a workplace had a greater effect on workplace health than improving lifestyle factors such as diet and exercise. This was illustrated through reduced feelings of stress, insecurity, and displacement for those workers who experienced a sense of belonging. Therefore, facilitating belonging within a workplace carries significant incentives for employers who wish to maximise the effectiveness and wellbeing of their employees (Thissen et al., 2023).

### Components of workplace belonging

Workplace belonging is significantly influenced by various components, including feeling valued and *fitting in* at work, connection levels and inclusion with colleagues (Waller, 2020), pride in the organisation and support for career development and daily tasks (Barton, 2023). A qualitative review by Sirkko et al. (2022) with school assistants revealed that belonging is heightened when there is strong trust and collaboration among staff, exemplified through regular meetings and diminished social hierarchies. Conversely, those with a lower sense of belonging reported unclear job roles and feelings of marginalisation, undervaluation, and burnout (Sirkko et al., 2022). Additionally, Belle et al. (2014) and Jasiński and Derbis (2023) found that supportive relationships with co-workers, which often leads to belonging, are linked with professional identity, happiness, and satisfaction. This body of work underscores the complexity of belonging and its critical role in the workplace, suggesting the need for a comprehensive and nuanced understanding of its determinants, particularly in educational settings where teachers are reporting unprecedented dissatisfaction globally (Zakariya et al., 2020).

### Belonging in education

The majority of belonging research in educational settings has centred on high school students (e.g., Allen et al., 2018; Korpershoek et al., 2019), with significant attention also given to post-secondary students (e.g., Allen et al., 2024; Crawford et al., 2023; Solanki et al., 2020; Tice et al., 2021). This focus has led to a notable gap in understanding belonging for others in educational contexts, including teachers. Additionally, despite the increasing research on teacher wellbeing (Collie & Martin, 2023; Vo et al., 2024), and the recognition of school belonging within an ecological system (e.g., Allen et al., 2021; Allen, Gallo Cordoba, et al., 2022), there remains a gap regarding understanding teachers’ sense of belonging. Addressing this omission is critical, as teachers play a fundamental role in shaping the educational environment and, consequently, the sense of belonging experienced by students and teachers alike (Uslu & Gizir, 2017; Wong et al., 2019).

### A critical emergency

Teacher attrition is an urgent global concern, leading to teacher shortages (Castro, 2022; Kelchtermans, 2017; Longmuir et al., 2022). Thus, several efforts have been made to understand factors that contribute to teacher attrition rates (Kelchtermans, 2017). Factors such as high workloads, a perceived lack of respect and support, curriculum limitations, little room for career progression, misalignment of personal and organisational values, and policy frustrations have been identified as contributing to the attrition of teachers (Kelchtermans, 2017; Longmuir et al., 2022; Madigan & Kim, 2021; Pillen et al., 2013). The attrition of qualified teachers concerns governments and schools alike (Castro, 2022; Kelchtermans, 2017). This shortage impacts student outcomes, increases existing staff workloads, and leads to financial and administrative challenges (Kelchtermans, 2017). From an organisational view, teacher loss affects cohesion and stability, adding to the burden on remaining staff (Castro, 2022; Kelchtermans, 2017). Research shows that a sense of belonging reduces attrition, suggesting that fostering teacher belonging may help address these shortages (De Neve & Devos, 2017; Thomas et al., 2019).

### Teacher belonging

Teachers who have a sense of belonging to school are more likely to have students who have a greater sense of belonging (Alonso-Tapia & Ruiz-Díaz, 2022; Bjorklund & Daly, 2021; Kachchhap & Horo, 2021). Research has also found that students achieve better outcomes when their teachers experience belonging (Gu & Day, 2013; Kachchhap & Horo, 2021), as belonging can lead to professional commitment (Kachchhap & Horo, 2021), self-efficacy (Bjorklund et al., 2020), and resilience (Bjorklund & Daly, 2021).

A recent report examining Australian teachers’ perspectives on their careers surveyed almost 5,500 respondents across various school types and locations (Longmuir et al., 2022). The findings revealed that while seven out of ten participants were intending to leave or considering leaving the field within the next ten years, eight out of ten teachers reported feeling they belonged to the profession. This relationship between belonging and retention warrants further investigation, particularly as research in other fields has linked a stronger sense of belonging to lower levels of attrition (Kelchtermans, 2017; Salles et al., 2019).

Longmuir et al. (2022) identified several factors that contributed to teachers’ sense of belonging, including quality relationships with colleagues and students, and feeling supported by the system and their schools. Conversely, factors that undermined belonging included feeling unappreciated or disrespected by staff, students, government, or society, and the burden of high workload. These competing experiences – such as experiencing quality relationships while struggling with high workloads – may help explain why teachers can simultaneously feel a sense of belonging yet consider leaving the profession. While most teachers reported feeling a sense of belonging, approximately 20% did not, showing a need for a deeper understanding of teachers’ sense of belonging to their profession.

### Current project

As previously noted, research on educational experiences of belonging is largely focused on the impact of belonging on student outcomes and not teachers. The current qualitative study aims to extend the work of Longmuir et al. (2022), by exploring facilitators of teachers’ sense of belonging. It is hypothesised that factors such as the perceived quality of relationships between colleagues, students, and leadership, a positive school culture, and feeling aligned with the requirements of their job will be associated with a sense of belonging for teachers.

## Method

### Participants

The sample consisted of 3,206 participants which included men (*n = *229), women (*n* = 2744), non-binary/gender diverse (*n = *13), and unlisted/prefer not to say (*n = *8). Career stages ranged from early career (0–5 years, *n = *572) to late career (*n = *846). While participants in this data set come from a range of educational backgrounds (early childhood -*n = *146-, primary education -*n = *1,238-, secondary education -*n = *958-, school leadership -*n = *489-, and other teaching fields -*n =  *163-, including tertiary educators, special education teachers, inclusive education teachers, or learning support teachers) they are referred to as teachers within this study. Further details on participant demographics can be found in the Appendix A see (Tables A1–A6).

Participants who selected “agree” and “strongly agree” to the statement “I feel a sense of belonging to the teaching profession” were included in the current study. The rationale behind selecting participants who already felt a sense of belonging in the teaching profession aligns with the study’s aim of understanding the key factors that cultivate this sense of belonging among teachers to provide a focused strength-based analysis of the positive influences and aspects of professional belonging.

### Materials

The original anonymous online survey, administered by Qualtrics Software, entailed a combination of Likert-style questions and extended response questions. These questions encapsulated topics relating to teacher’s perspectives on their profession, such as respect, appreciation, job satisfaction, belonging, retention, attrition, and working conditions. For this study, a sense of belonging was examined through participant responses to the open-ended questions, “What has a positive influence on your sense of belonging to the teaching profession?” and “What has a negative influence on your sense of belonging to the teaching profession?”

### Procedure

Participants accessed the survey through an online link and could complete the survey at their convenience between 9 May and 30 May 2022. Participation invitations were sent out via the original project team’s professional networks, social media (Facebook, Twitter, and LinkedIn), and the University’s alumni networks and social media (Facebook, Instagram, LinkedIn, and Twitter). Ethics approval was granted by Monash University’s Human Research Ethics Committee (32494).

### Analysis

A qualitative paradigm was used, using thematic analysis (TA). In line with the methodology of Braun and Clarke (2019), TA involves a six-phase analysis to identify themes within survey data. The initial phase of the analysis entailed thorough data familiarisation, achieved by a comprehensive reading of the dataset. Subsequently, in the second phase, initial coding was independently conducted by two researchers (MT & KA), in line with Braun and Clarke’s (2019) thematic analysis framework. This step ensured an unbiased approach to identifying preliminary themes. Discrepancies between the researchers’ interpretations were systematically addressed through discussion, ensuring a consensus was reached on the emergent themes. The subsequent phases focused on the review and refinement of these themes, culminating in the overarching themes of the dataset. The full authorship team then critically evaluated the final themes and subthemes to ensure they accurately represented the data.

## Results

### Themes influencing teacher belonging

Six core themes and their respective subthemes were identified as factors that contribute to teachers’ sense of belonging. These core themes include: Interpersonal relationships (peer relationships, student and parent relationships); Support and collaboration (providing and receiving support; team dynamics and collaborative efforts); Professional and personal growth (influence and contribution to society, professional learning opportunities, individual professional identity and characteristics); Institutional factors (leadership support, positive school environment, employment stability), Motivators (teaching passion, acknowledgement and appreciation); and External networks (engagement in professional networks).

**Figure 1.**
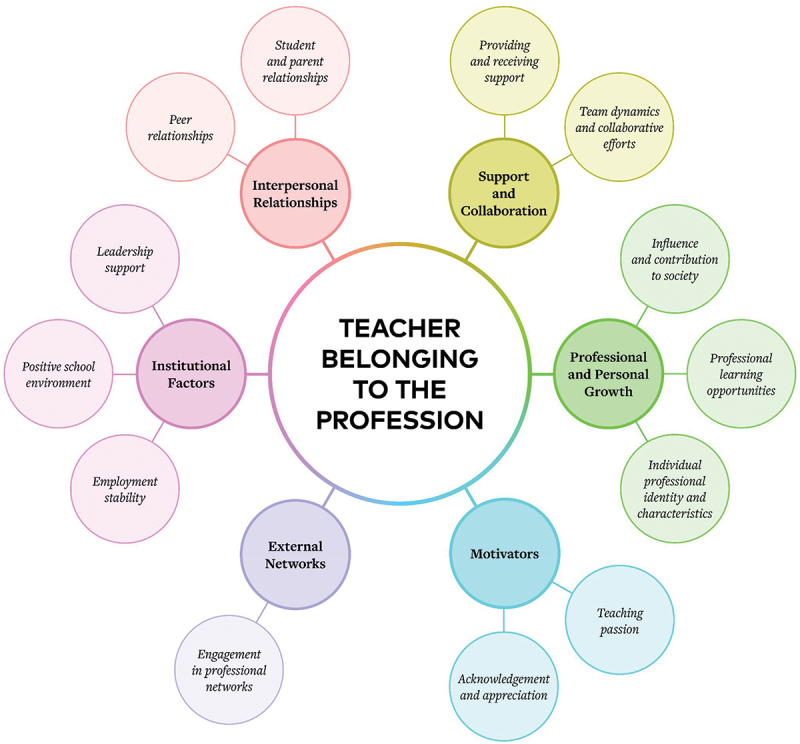
Key influences on teacher belonging to the profession.

Each of these themes and subthemes collectively show the multifaceted influences on teachers’ sense of belonging to the profession (Figure 1). We note here that these thematic categorisations are presented as discrete for the purpose of the study but acknowledge that there are many overlaps and interactions within and across the themes and subthemes.

### Interpersonal relationships

#### Peer relationships

Relationships with co-workers emerged as a prominent theme, mentioned by 75.32% of teacher responses. Teachers reported that connections with workplace friends and peers fostered growth, professional development, collaboration, unity, camaraderie, and teamwork – all of which enhanced belonging. These relationships enabled teachers to share both positive and negative teaching experiences, as well as their passions and reasons for choosing the profession. Peer interactions provided opportunities for professional dialogue and workload support, as one teacher noted: “support for each other when the workload is high”. Teachers emphasised that they “are the only ones who understand the frustration and workload, so we have a common bond. Teachers can ‘talk shop’ from school to school and ‘get it’ – sharing ideas and frustrations”.

Shared experiences with colleagues particularly strengthened teachers’ sense of belonging. Teachers valued their “shared frustrations … shared burdens” and appreciated that “everyone is experiencing the same feelings, understands the same terms (jargon), … speaks the same language and understands each other”. One teacher explained: “Only teachers understand the day in the life of a teacher. There’s nothing better than a debrief with your team at the end of the day. We really are in this together and we all work together to support our kids. We all have a common goal and that is to support our kids to achieve all that they can. The common goal creates a sense of belonging to the teaching profession”.

While sharing challenging aspects of teaching (such as stress) was frequently mentioned, teachers also emphasized the importance of celebrating successes and working with like-minded peers. Additionally, some teachers noted that having immediate family members in the profession enhanced their sense of belonging.

#### Student and parent relationships

Relationships with students (21.64% of responses) and parents (6.11% of responses) were also reported to have a role in fostering teachers’ sense of belonging. Teachers reported that parental appreciation and trust enhanced their sense of belonging. Long-term connections were particularly meaningful, including watching “cohorts of kids through to matriculation and beyond”, teaching multiple siblings from the same family, and “building connections with students and their families”. As one teacher noted, “the kids and their siblings, I know them all and watch them grow up over the years”.

Teachers also found belonging through witnessing their students’ success and growth, particularly through “seeing the effects of my actions bettering the lives of families and students in the community”. Their sense of belonging was further strengthened when students recognised them both inside and outside of school, and through “creating a class family each year and making memories” with their students.

### Support and collaboration

#### Providing and receiving support

Teachers reported that both receiving and providing support strengthened their sense of belonging, with this theme appearing in 12.63% of responses. While most support came from colleagues (90.19% of responses), it also came from leadership, parents, and students.

Support encompassed mental, emotional, practical, and workload assistance. Teachers valued when “executive staff do what they can to reduce work and stress loads”. Important support activities included “being able to talk about issues and concerns” and “sharing resources, debriefing, or offering advice”. One participant noted that their “Head Teacher promotes honest sharing in the staffroom”, which helped them feel less “alone in (their) struggles”.

The collegial nature of teaching was highly valued, mentioned by 6.41% of respondents, with unity and shared responsibilities being crucial for belonging. Reciprocity emerged as a key element of this support, exemplified by one teacher’s comment about “Providing assistance to other teachers and receiving it in return”.

#### Team dynamics and collaborative efforts

Another theme that emerged was teamwork and collaboration, noted by 9.35% of respondents. Teachers highlighted the importance of strong effective teamwork as a facilitator to their sense of belonging. This related to collaboration with other teachers as well as participation in formal teams such as specific faculties. “Working with other teachers in a team to achieve a common goal”, and planning and sharing of ideas and resources were commonly raised as enjoyable outcomes of teamwork associated with belonging. For instance, “I work in a really supportive team right now… We have each other’s backs. We know our batteries aren’t always at 100% and we give more when others can’t give their all”.

Strong communication between team members kept teachers up to date and involved in matters important to their work. Additionally, feeling trusted by team members and being able to share ideas or concerns in a non-judgemental context created a higher quality team environment. These features of team dynamics fostered a greater sense of belonging for respondents.

### Professional and personal growth

#### Influence and contribution to society

A further theme highlighted by 7.25% of respondents was their perceived impact on society. Teachers expressed that their sense of belonging was strengthened by their belief in the value of their work and their impact on young people and society. The belief “that education is fundamental to a functioning, flourishing society” was present throughout this theme. The impact of their work extended from small “aha moments” in the classroom (where students are observed to express the understanding of a new concept) to watching students grow, develop, and succeed over time and into the future. When learning reached beyond the classroom to influence students’ families and communities, teachers experienced a stronger sense of belonging and professional satisfaction. A strong overriding motif was the “ability to connect curriculum outcomes to student development on their path to adulthood”. One respondent suggested: “I am helping shape our next generation of politicians, nurses, child care workers, stay at home parents, scientists etc. and each of them are equally important in shaping the next generation”.

#### Professional learning opportunities

Professional development (PD) and the opportunity to build on their knowledge and pedagogy was identified in 4.51% of teachers’ responses. Teachers remarked that PD provided numerous benefits, such as the opportunity to broaden their skills and knowledge while also networking with colleagues and peers. PD also facilitated collaboration and information sharing between schools and allowed teachers to reconnect with previous colleagues. One participant remarked their sense of belonging derived from the “opportunity to network with others through professional development” but that “this has reduced in the past ten years” citing budget and time considerations. Notably, participants felt that online methods of PD delivery were less preferred due to the difficulties of stimulating organic connections between peers as it “doesn’t allow for the collegial discussions and support during breaks/networking”.

While school-wide PD was appreciated by respondents, they felt that their sense of belonging was better supported when they had the autonomy and flexibility to choose their own PD as this supported their personal interests and needs. Further, being able to share their learning and interests with colleagues also facilitated belonging. For example, one participant mentioned:

I have a strong love of learning and I seek out new ways to engage with recent research to improve my teaching practice. Sharing this knowledge and working with different staff at my school gives me this sense of belonging. We are working together to improve our practice that will benefit our students.

Being mentored by and mentoring other teachers was also reported as a facilitator of belonging. Teachers reported that mentors contributed to their professional learning and supported them with their workload. Mentors provided encouragement and reinforcement. Likewise, “being asked to mentor teachers at many levels” promoted the sense that their “expertise is valued and sought after for helping others”, which provided validation of teachers’ own contributions to the profession and fostered their sense of belonging. Mentors were important to teachers early in their careers, and experienced teachers valued the additional role of mentoring less experienced teachers.

#### Individual professional identity and characteristics

Skills and personal characteristics of teachers emerged as another common theme. Of the surveyed teachers, 3.54% reported a personal ability to educate students. This encompassed factors such as having strong knowledge of their chosen teaching area or subject and feeling skilled as a teacher in general. For example, one teacher reported “I am a specialist mathematics teacher and I know my content thoroughly. I realise this makes me a valuable maths teacher”.

Teachers indicated that having “a natural ability” or experiencing a “good fit of skills” for teaching promoted their sense of belonging. Furthermore, personal characteristics such as mindset, work ethic, personality, and “traits that align directly with other people in the teaching profession” were also highlighted.

Some teachers noted their sense of belonging was associated with experiencing an affinity to the profession and their identity as a teacher. Teachers reported that school was where they “belonged and could be (themselves)” and that they were “called to be” teachers, suggesting that taking pride in their work and status as a teacher facilitated belonging. Having a teacher identity was linked to the number of years spent in the profession, with 1.57% of teachers indicating that their “years of experience” helped them feel as though they belonged. Longer service in the profession provided more opportunities to build connections with their school community and develop relationships with other staff members. This also included experiencing different educational contexts throughout their career.

### Institutional factors

#### Leadership support

The theme of leadership was present in 6.11% of responses. Notably, a sense of belonging was experienced when teachers felt that they were supported by their middle and upper leadership teams. Leaders who understood the difficulties teachers experienced were valued, and teachers reported that they appreciated it when leadership continued to teach classes, as this maintained their relatability and kept their goals for staff realistic. One teacher noted that “management having a meaningful teaching load, so they understand what it’s like in the trenches” facilitated their sense of belonging.

Teachers reported that they felt they belonged when leadership allowed them to be “autonomous”, use their professional judgement, creativity, and ideas, rather than enforcing their own ideals of teaching. For example, one teacher described that “the school supporting me and being able to have ownership over my decision and classroom” promoted their sense of belonging. Being involved in decision-making related to their roles and being kept informed of decisions were also highlighted as important for their sense of belonging. Leadership also played an important role in supporting teachers’ wellbeing, “allow(ance) for capacity building”, and setting realistic goals. Teachers reported that support from leadership varied between schools and was often not as robust as they would like. For example, one teacher responded: “I am lucky and have a kind and understanding executive team. I have not always been so lucky”.

#### Positive school environment

Positive school culture emerged as a theme, with 5.61% of teachers reporting that being part of a “close-knit” community with positive staff mentality promoted belonging. Daily practices like morning tea breaks, end-of-term celebrations, and professional development days helped create this sense of belonging. Social gatherings, such as “Friday afternoon drinks” and other events throughout the semester, fostered welcoming staffroom environments. Teachers noted that these activities created inclusive community atmospheres and enjoyable school cultures. As one teacher explained, “We have a social club that organises staff activities for our staff to bond and have fun together. It really makes your workplace a nice environment”. Additionally, teachers reported stronger belonging in schools with a clear ethos and purpose. Specifically, schools with a “clear sense of direction” and “common goals” had practical, positive impacts on teachers’ sense of belonging. For instance, some teachers noted that working within faith-based schools united staff through shared principles.

#### Employment stability

Employment contract type and duration emerged as another theme. A small percentage (0.5%) of teachers reported that fixed-term contracts requiring annual reapplication hindered their sense of belonging. These short-term contracts created what teachers described as “a financial nightmare” and feelings of instability. The repeated interview process was called “disheartening”, further challenging teachers’ professional belonging. In contrast, teachers who were “lucky enough to have an ongoing contract” reported stronger connections to their schools and a greater sense of belonging. This “permanence to their school” enabled teachers to better plan for their students and invest in programs and relationships. Even simple organisational provisions, such as a “pigeon hole” for casual staff to store their items, helped foster a sense of belonging.

### Motivators

#### Teaching passion

Passion for teaching emerged as another recurring theme, appearing in 6.68% of responses. Teachers reported multiple sources of this passion: the teaching process, specific subjects, working with children, student welfare, quality education, guiding the “next generations”, public education, and learning. Many described it as a “privilege to have a formational role in a child’s life”. Teachers consistently noted that their passion for helping students and children fostered a sense of belonging. This feeling was strengthened by working alongside “amazing peers who share my passion for teaching, learning & people”, allowing teachers to share their enthusiasm with colleagues. Teachers also noted that witnessing the “hard work” of colleagues “who are willing to bust their guts to make it better for kids” enhanced their sense of belonging.

#### Acknowledgement and appreciation

Receiving positive feedback and recognition reinforced teachers’ sense of professional belonging, similar to receiving support. This theme of feeling appreciated appeared in 5.44% of responses. Words of appreciation, gratitude, and praise from colleagues, leadership, students, and parents strengthened teachers’ sense of belonging to the profession. A key component was having their successes recognised and receiving thanks for their work. As one teacher noted, “I have had great feedback from my students and their parents which reassured me I’m doing well”. Teachers frequently mentioned that appreciation of their hard work from students, leadership, and parents made them feel “valued” and validated in their roles. Being respected and accepted as a professional by students, parents, colleagues, the community, and leadership also promoted belonging. One teacher described this experience: “When I tell people in my community, I am a teacher they trust me more, they smile, they relate to me. In general people understand working with young people is difficult and they are grateful I do this”. Recognition and respect from leadership specifically reinforced that teachers were performing well and made them feel “valued and supported”.

### External networks

#### Engagement in professional networks

Beyond the immediate school environment, 2.37% of teachers reported that participation in professional organisations and online communities (including social media) influenced their sense of belonging. These networks helped reduce teacher isolation and encouraged active participation in the profession. For instance, one respondent noted that serving as a union representative enhanced their sense of belonging by enabling them to improve working conditions for fellow teachers.

Teacher-specific social media and online communities also contributed to belonging by facilitating connections with peers and providing access to “the support and friendship of teachers from around the world”. These platforms enabled teachers to stay connected, share professional learning and resources, exchange experiences, and find like-minded colleagues with similar interests (such as specific subject areas). They also created spaces where teachers could support each other through both successes and challenges. Through these various functions, professional organizations and online communities strengthened teachers’ sense of belonging.

## Discussion

This study aimed to identify factors that facilitate Australian teachers’ sense of belonging to their profession. This study identified six key themes that facilitate Australian teachers’ sense of belonging: Interpersonal relationships (peer relationships, student and parent relationships); Support and collaboration (providing and receiving support; team dynamics and collaborative efforts); Professional and personal growth (influence and contribution to society, professional learning opportunities, individual professional identity and characteristics); Institutional factors (leadership support, positive school environment, employment stability), Motivators (teaching passion, acknowledgement and appreciation); and External networks (engagement in professional networks).

### Interpersonal relationships

The current study highlights the significant role of colleagues and peers in fostering teachers’ sense of belonging. Positive relationships with colleagues, students, and parents were the most cited facilitators, aligning with existing research on the importance of relationships for belonging (Barton, 2023; Kuurne & Vieno, 2021; Lambert et al., 2013; Longmuir et al., 2022; Melzak et al., 2024; Skott, 2018). Relationships with colleagues were seen as central to professional growth, collaboration, and support. This supports theoretical perspectives on belonging (Baumeister & Leary, 1995; Maslow, 1943; Ryan & Deci, 2000), which emphasise relational factors.

Parents and students provided a source of support and encouragement for teachers’ work, which has been associated with belonging (Lambert et al., 2013). While relationships with both students and parents were important, students played a larger role in teachers’ sense of belonging. This is likely due to the more frequent and direct interaction teachers have with students. Positive student relationships, academic success, and a sense of community in the classroom were identified as vital to fostering belonging. Although the specific mechanisms linking academic success to belonging were unclear, previous research suggests that teachers’ professional identity, which is closely tied to student success, contributes to a stronger sense of belonging (Beauchamp & Thomas, 2009; Clandinin et al., 2015).

### Support and collaboration

The collegial relationships outlined in the previous theme underpin other facilitators, such as teamwork, collaboration, and peer support. Teaching, inherently a collaborative profession, thrives on collegiality and teamwork (Banerjee et al., 2017; Valckx et al., 2019; Vangrieken et al., 2017), with a supportive work environment enhancing belonging through acceptance and peer connections (Morettini et al., 2020; Thissen et al., 2023). Given the collaborative nature of teaching, it is unsurprising that Australian teachers often report strong professional belonging (Longmuir et al., 2022). This is consistent with tertiary teachers, with a similar study finding that a culture of “co-caring” and collaboration strengthened their sense of belonging (Joseph et al., 2021). A supportive and collegial environment that fosters belonging is promoted by supportive leadership, a finding from this study that aligns with previous research (Banerjee et al., 2017; Mason & Matas, 2015; Pendergast et al., 2020).

### Professional and personal growth

In the current study, teachers felt a sense of belonging when their work contributed positively to society and aligned with their professional teaching identity. Teachers in this study expressed pride in the academic and developmental value of their teaching, and this sense of contributing meaningfully to society was linked to their belonging, similar to previous research (Alexander et al., 2020; Barton, 2023). The desire to make a social impact and influence future generations, a key motivator for teachers (Alexander et al., 2020), also reinforces their sense of belonging to the profession. These findings align with studies on value consonance and belonging (Bjorklund & Daly, 2021; Skaalvik & Skaalvik, 2011), suggesting that when teachers’ work matches their professional identity and societal contributions, they feel a stronger connection to the teaching profession. Leadership can also play a role here, with teachers in this study reporting that leadership impacted school ethos and identity.

The findings suggest that teachers feel a sense of belonging when their school has a clear, positive goal, ethos, and identity. This was expected, considering that individuals who feel pride and alignment with their workplace’s values and purpose have been found to be more likely to experience belonging (Barton, 2023; Belle et al., 2014). Additionally, teaching identity and abilities contributed to their sense of belonging. Confidence in their teaching skills was identified as a key factor in fostering belonging. This contrasts with Skaalvik and Skaalvik’s (2019) study, which found no direct link between self-efficacy and belonging, though they noted relationships with other factors, such as resources and job demands. Further research is needed to explore the connection between self-efficacy and belonging among teachers.

Mentoring emerged as a key factor in fostering teachers’ sense of belonging. As a common method of professional development (Ambler et al., 2016), mentoring promotes belonging by encouraging participation, collaboration, and engagement (Naidoo et al., 2021; Naidoo et al., 2022. The data revealed mentoring to be important for promoting belonging, for both the mentor and the mentee, as it provided role models, continued skill development, and support. Importantly, the investment in mentoring also indicates value for the time and expertise of teachers, with this indication of appreciation supporting a sense of belonging (Naidoo et al., 2021). Informal staff interactions, such as morning teas, were also important for sharing experiences and strengthening connections.

Professional development was identified as important to facilitating a sense of belonging. In-person PD allowed for the development of collegial relationships and collaboration that can foster a sense of belonging via networking, sharing of teaching practices and experiences, and the opportunity for teachers to source knowledge from outside their schools. PD and being mentored promoted the ability of teachers to continue to develop their pedagogy, promoting their connection and sense of belonging to the profession. Existing research has extensively demonstrated the importance of PD for promoting high-quality teachers, which has implications for student outcomes (Kalinowski et al., 2019; Sancar et al., 2021), however little research has specifically examined its impact on teacher belonging.

### Institutional factors

Leadership plays a key role in fostering teacher belonging and is linked to multiple themes such as developing a supportive school environment and assisting personal and professional growth. Consistent with previous research, this study found that leadership approaches significantly impact teachers’ sense of belonging by either fostering or hindering collaboration, autonomy, and trust (Mason & Matas, 2015). Teachers in this study emphasised the importance of being trusted to exercise professional autonomy, which aligns with existing literature that links teacher autonomy with belonging and retention (Kelchtermans, 2017; Sirkko et al., 2022). Autonomy, when supported by trust, empowers teachers, bolstering their professional identity and sense of competence, both of which are associated with stronger belonging to the teaching profession (Pendergast et al., 2020).

However, this study also highlighted the potential drawbacks of autonomy in high-accountability environments, where teacher performance is closely linked to student outcomes. In these contexts, high levels of autonomy can become isolating, as teachers may feel compelled to work individually to avoid criticism, undermining collaboration (Valckx et al., 2019; Vangrieken et al., 2017). This contrasts with the findings of this study, where teachers consistently reported that collaboration and feedback, were vital to their sense of belonging. The tension between autonomy and collaboration observed here reflects concerns raised in the literature about the impact of increased teacher autonomy in competitive, high-stakes environments (Ormond, 2016; Salokangas et al., 2019).

Teachers in this study, like those in previous research, felt that a supportive school ethos – fostering trust and collaboration – was essential for professional growth and a positive teaching identity. This highlights the need for a balanced approach to autonomy, one that aligns with teachers’ need for collaborative support and shared professional development, as this balance promotes a stronger, more sustainable sense of belonging in the teaching profession. Moreover, where teachers are trusted, providing autonomy suggests greater belief in their teaching ability and solidifies their teaching identity (Pendergast et al., 2020). This personal belief in teaching identity was found to promote a sense of belonging in this study.

Job stability emerged as a key factor in fostering teacher belonging, with some teachers noting that casual contracts negatively affected their sense of connection to the school community. Casual contracts are common in Australia and have been identified as a concern for teachers (Brown et al., 2013; Crimmins, 2016; Reupert et al., 2023; Uchida et al., 2020). Research shows that teachers on casual contracts are less likely to participate in school events, professional development, and mentoring, and have fewer opportunities to build relationships with colleagues and students (Brown et al., 2013; Crimmins, 2016; Uchida et al., 2020). They also face isolation, limited access to school resources, and are less likely to be recognised for their work or involved in collaborative planning (Brown et al., 2013). While few teachers in this study raised casual contracts as a concern for belonging, this may reflect the study’s inclusion of teachers who already felt a sense of belonging. Future research could explore how casual contracts impact teachers who do not experience belonging. Given the increasing prevalence of casual employment in Australia (Australian Bureau of Statistics, 2022), these findings are relevant for future strategies aimed at promoting belonging and reducing attrition among teachers on casual contracts.

### Motivators

This study found that a sense of belonging enhanced the passion teachers had for their profession. This was expected considering the importance of shared experiences and goals in promoting workplace belonging and its link to the development of mutual appreciation of the objective, purpose, and benefits of the work (Barton, 2023; Lampinen et al., 2018).

Sense of belonging was also found to stem from acknowledgement and appreciation of teachers’ work. Likewise, it was important for teachers to feel valued and respected by their leadership teams. This is consistent with the workplace belonging literature where recognising accomplishments and contributions can foster a sense of belonging (Ruedas-Gracia et al., 2022).

### External networks

Professional organisations and unions were identified as important for some teachers’ sense of belonging. Professional organisations have been identified as a powerful resource for career development as they provide networks for individuals to explore their chosen skills, subjects, and interests (Owen, 2016; Soito & Jankowski, 2023). These organisations offer long-term engagement opportunities that are more beneficial than one-off professional development (Owen, 2016), fostering collaboration and personal learning within professional learning communities, which are linked to reduced burnout and increased well-being (Owen, 2016; Soito & Jankowski, 2023). In this study, teachers also recognised union membership as important for their sense of belonging, although little research has explored this connection. While unions are known to offer benefits like higher pay, reduced stress, and improved well-being (Han, 2019; Han & García, 2022), their direct role in fostering belonging and reducing attrition remains underexplored. It is possible that unions provide a collective voice for teachers, which could contribute to belonging, but this needs further investigation (Han, 2019). Nonetheless, the results of this study suggest union and professional organisation membership may be facilitators of teachers’ sense of belonging. It is important to note that memberships to unions and professional organisations are generally associated with financial and time barriers which may prevent some teachers from accessing a resource that may otherwise benefit them (Gavin et al., 2020; Owen, 2016).

In addition, social media and online communities were noted as being facilitators of belonging. Platforms such as Instagram have become centres for teachers to communicate, support one another, share ideas and knowledge, and develop their pedagogies outside of formal PD (Newton & Williams, 2021). Social media can provide teachers with resources and professional networking in a timely and cost-efficient manner; however, it has also been used to promote financial gains for some users (Davis & Yi, 2022). For example, some social media platforms are used to market teachers’ products or services to other teachers (Davis & Yi, 2022). Therefore, caution should be taken to avoid financially exploiting teachers when using social media to promote belonging, however, it may act as a valuable, time-effective resource if used correctly to support retention.

### Limitations

This study has several important limitations to consider. First, since the data came from a larger project examining various aspects of teachers’ work (including job satisfaction, safety, and working conditions), analysing only the belonging-related responses may not capture the full complexity of teachers’ experiences. While our findings provide valuable insights, a study specifically designed to examine professional belonging would be beneficial to extend this work.

Second, the study’s broad focus on teachers as a general population means it may not adequately represent the experiences of specialised teaching groups, such as special education teachers. The findings should therefore be applied cautiously to these specific teaching populations.

Third, an important conceptual limitation emerged around the distinction between belonging to the teaching profession versus belonging to a specific workplace. Many factors identified by participants (such as positive school environment) were workplace-specific and may not directly translate to professional belonging more broadly. Future research needs to carefully examine this distinction and investigate how workplace and professional belonging interact.

### Implications and future directions

The findings of this study contribute to a greater understanding of the factors that facilitate Australian teachers’ sense of belonging to the profession. These findings are particularly relevant to priority area five of the Australian Government’s National Teacher Workforce Action Plan (AGNTWAP), which outlines a need for increased collection of data regarding teacher wellbeing (Australian Government Department of Education, 2022). While this research does not directly contribute to the AGNTWAP, the greater understanding of the facilitators of teachers’ sense of belonging provided by this research increases the literature pool of information available to develop strategies that target teacher wellbeing and prevent attrition. The findings of this research suggest that factors such as social support, collegial relationships and experiencing a positive school culture are especially important for increasing a sense of belonging for teachers, which has the potential to promote teacher wellbeing and reduce attrition (Barton, 2023; Belle et al., 2014; Kelchtermans, 2017).

## Conclusion

Overall, the findings of this study largely confirmed the hypothesis that factors such as the relationships between colleagues, students, and leadership, and a positive school culture, facilitate teachers’ sense of belonging. This study further reinforces the integral role of relationships with colleagues, peers, leadership, students, and parents in facilitating belonging to the teaching profession. Future research into the barriers to teachers’ sense of belonging is needed to better understand sense of belonging within the teaching profession. Overall, strategies to facilitate a sense of belonging may support teacher wellbeing, and the retention of quality teachers in the field.

## Appendix A

**Table A1.** Percentage of school teachers in Australian school sectors.

School Type				
Primary School	Secondary School	School Leader	Early Childhood	Other
43.2	33.43	14.13	4.32	4.92

**Table A2.** Number of participants by gender.

Gender	Number of Participants
Woman	2,745
Man	229
Non-Binary/Other	10
Not Listed	2
Prefer Not to Say	8
Total	2,994

**Table A3.** Number of teachers by years in profession.

Years in Profession						
<1	1–3	4–5	6–9	10–19	20–29	30+
70	231	271	609	967	563	283

**Table A4.** Number of teachers per Australian State/Territory of residence.

State/Territory							
NSW	VIC	QLD	WA	SA	TAS	NT	ACT
700	1106	531	307	162	64	33	91

**Table A5.** Teachers by location.

Location				
Inner Suburb	Outer Suburb	Regional City	Rural Area	Remote Area
736	1154	679	384	41

**Table A6.** Teachers by age group (in years).

Age					
20–29	30–39	40–49	50–59	60–69	70+
456	1003	803	635	93	4
